# Effect of chronic stress on gel-forming mucins in the small intestine of BALB/c mice

**DOI:** 10.25122/jml-2023-0473

**Published:** 2024-03

**Authors:** Jennifer Karume Gutiérrez-Galicia, Maria Elisa Drago-Serrano, Rigoberto Oros-Pantoja, Marycarmen Godínez-Victoria, Fabiola Guzmán-Mejía

**Affiliations:** 1 Sección de Estudios de Posgrado e Investigación, Escuela Superior de Medicina, Instituto Politécnico Nacional, Ciudad de México, México; 2 Departamento de Sistemas Biológicos, Universidad Autónoma Metropolitana Unidad Xochimilco, Ciudad de México, México; 3 Facultad de Medicina, Universidad Autónoma del Estado de México, Toluca de Lerdo, México

**Keywords:** immobilization stress, alcian blue, mucin 2, mucin 5AC, interleukin-18, IgA–microbiota complexes

## Abstract

Intestinal homeostasis involves the collaboration of gut barrier components, such as goblet cells and IgA–microbiota complexes, that are under the control of stress that promotes inflammatory responses addressed primarily in the colon. The aim of this study was to evaluate the effect of stress on mucins, goblet cells, and proinflammatory parameters in the proximal and distal regions of the small intestine. A group (*n* = 6) of female 8-week-old BALB/c mice underwent board immobilization stress (2 h per day for 4 days) and were sacrificed with isoflurane. Samples from proximal and distal small segments were collected to analyze the following: 1) goblet cells stained with periodic acid-Schiff (PAS) and with alcian blue (AB) to visualize histologically neutral and acidic mucins, respectively; 2) IgA–microbiota complexes identified by flow cytometry in intestinal lavages; and 3) *MUC2, MUC5AC*, and *IL-18* mRNA levels in whole mucosal scrapings by reverse transcription–qPCR. Regarding the unstressed group, in the proximal region of small intestine both PAS+ and AB+ goblet cells were unchanged; however, *MUC5AC* and *IL-18* mRNA levels were increased, and the percentage of IgA–microbiota complexes was reduced. In the distal segment, the number of PAS+ goblet cells was increased, whereas the number of AB+ goblet cells was reduced and did not affect the remaining parameters. The data suggest that stress induces inflammation in the proximal small intestine; these findings may provide an experimental reference for human diseases that may affect the proximal small intestine, such as Crohn’s disease, in which stress contributes to the progression of intestinal inflammation or relapse.

## INTRODUCTION

The intestinal barrier helps maintain intestinal homeostasis by allowing the selective entry of molecules through the epithelial monolayer to the inner milieu [1]. Proper functioning of the intestinal barrier entails the mucus layer that overlies the luminal surface of the epithelial monolayer; mucus blocks direct contact between the microbiota and the epithelial cell surface to prevent potential inflammatory responses resulting from signaling pathways triggered by the interaction of microbial molecules with innate receptors [2]. Mucus is secreted by goblet cells, whose number is greater in the colon than in the small intestine [3]. Mucus is composed of gel-forming mucins, such as mucin 2 (MUC2), which is secreted in the small intestine and colon, and MUC5AC, which is secreted prominently in the stomach [4]. In the proximal small intestine, mucus is present as a monolayer loosely attached to the epithelial surface. In the colon, mucus comprises a bilayer, one of which is firmly attached to the epithelial surface and covered by a loosely attached monolayer that directly contacts the luminal microbiota [4]. Mucus acts as a sticky matrix in which immunoglobulin A (IgA), an anti-inflammatory effector of mucosal immunity, is secreted [2,4]. Both IgA and the microbiota are more abundant in the colon than in the small intestine; these components form IgA–microbiota complexes that favor luminal clearance to ameliorate potential inflammatory responses [5–7]. In fact, IgA–microbiota complex levels are increased in some diseases, such as diarrhea-predominant irritable bowel syndrome [8]. The intestinal epithelial monolayer has different functions and compositions, as well as divergent neuroendocrine frameworks in the small intestine and colon [9]. Growing evidence has shown that stress disrupts the interactions of microbiota with mucus, favoring inflammatory conditions in the intestinal environment through the activation of signaling pathways, thus resulting in the release of stress hormones [10]. Unlike the large number of studies focused prominently on the colon, only a few assays based on a murine model of restraint stress [11–13] have analyzed mucin production and goblet cell numbers in the proximal and distal small intestine in response to stress. This approach may unveil the underlying mechanisms through which stress alters the function or structure of goblet cells and mucins in each segment of the small intestine. In this study, we aimed to evaluate gel-forming MUC2 and MUC5AC and goblet cells in terms of regionalization of the small intestine in mice subjected to chronic immobilization stress. The data suggest that stress induces inflammation in the proximal small intestine; these findings may provide an experimental reference for human diseases that may affect the proximal small intestine, such as Crohn’s disease, in which stress contributes to the progression of intestinal inflammation or relapse.

## MATERIAL AND METHODS

### Animals

Groups of 6-week-old female BALB/c mice were obtained from the Unidad de Producción y Experimentación de Animales de Laboratorio (UPEAL), Universidad Autónoma Metropolitana Unidad Xochimilco (UAM-X). Mice were adapted to the environmental conditions for 2 weeks and housed in two groups (control and stress) with six animals per cage at 22–24 °C and with 55% relative humidity and a 12-h light/dark cycle (7:00 a.m./7:00 p.m.). The animals were fed a rodent diet (Laboratory Rodent Diet 5001, LabDiet) and purified water ad libitum. Mice were cared for and handled according to the Mexican Federal Regulations on Animal Experimentation and Care (NOM-062-ZOO-1999) and the Ministry of Agriculture, Mexico City, Mexico, following the established norms of the Organization and Procedures of the Internal Committee for the Care and Use of Laboratory Animals from UAM-X. This protocol was approved by the CICUAL-UAM-X under register number 176 (DCBS.CICUAL.007.2020).

### Immobilization stress protocol

The protocol for board-immobilization stress was conducted as previously described in detail [14]. In brief, at 8 weeks of age, mice (*n* = 6) were immobilized on an expanded polystyrene board in the prone position, holding all four limbs and the tail with duct tape. First, the front legs were immobilized, then the pads of the hind legs and, finally, the middle part of the tail. Cardboard strips, like chewers, were placed over adhesive tape to prevent self-inflicted injuries to the skin on the front leg. At the time of the trial, free movement of each mouse’s head was allowed, and whisker tearing was avoided. At the end of the 2-h immobilization test, the adhesive tape was removed in the following order: tail, hind legs, and front legs. The model was repeated daily for 4 days, starting between 8:00 and 11:00 a.m. At the same time, the control group (*n* = 6) was deprived of water and food during the same period as the mice that were subjected to immobilization stress. Two independent assays (*n* = 6 per group per assay) were performed.

### Biological samples

After the last period of stress, both groups of mice were weighed and subsequently sacrificed via exposure to 300 µl of inhaled isoflurane in a glass container. After that, the skin from the abdominal region was cleaned with benzalkonium chloride and cut, the peritoneal membrane was exposed, and the small intestine was dissected. The reference points of the small intestine were 0.2 cm below the pylorus and 0.2 cm above the cecum. Subsequently, the small intestine was divided into two equal parts to obtain the proximal and distal segments. Each intestinal segment was washed with 1 ml of PBS, pH 7.4, and intestinal lavage was used to identify the IgA–microbiota complex by flow cytometry. Then, the intestine was everted and longitudinally cut to expose the mucosa, which was scraped off with a glass slide and placed in TRIzol to determine the gene expression of mucins by reverse transcription–qPCR (RT‒qPCR). Total tissue from each intestinal segment was stored in 10% formol PBS for histological analysis.

### Histological analysis of goblet cells

Samples from both intestinal segments were fixed in 10% formol PBS for 48 h, washed twice with 70% ethanol and then embedded in paraffin for periodic acid-Schiff (PAS) staining to detect neutral mucins and alcian blue (AB) staining to observe acid mucins according to a previously described procedure with some modifications [14].

For histological analysis, the samples were cut into 6 µm thick slices, deparaffinized with xylene, rehydrated gradually by immersion in solutions with a decreased ethanol concentration and incubated in 0.5% PAS for 15 min. Afterward, the samples were washed with distilled water, tap water and PBS, incubated with Schiff’s reagent for 10 min and then washed again as before. For AB staining, the samples were washed with 3% glacial acetic acid for 3 min, stained with AB solution for 2 h, washed, and subsequently transferred to 3% glacial acetic acid for 3 min. Both the AB and PAS samples were stained with Harris hematoxylin as a contrast dye for 5 and 10 min, respectively, before histological observation.

The cell morphology was visualized via light microscopy (Axiostar Plus, Carl Zeiss) using Lumenera Infinity Analyze and Capture software v.6.5.4 (Lumenera, Microsoft Windows 10). To measure the size and number of goblet cells, images were analyzed using Image-Pro Plus v.7.0 software (Media Cybernetics, Microsoft Windows 10).

### Analysis of the IgA–microbiota complex

The analysis of IgA–microbiota complexes was based on a modified protocol [15]. In brief, intestinal lavages in PBS were centrifuged at room temperature and 9.2×g for 5 min. The pellet was recovered, suspended in 500 µl of thioglycolate broth (BD Bioxon, BD Biosciences) and stored at −20 °C until analysis. For analysis, the samples were suspended in 2 ml of PBS, homogenized and filtered through a 100 µm-pore nylon membrane to remove fecal aggregates from bacteria. After that, the samples were washed by suspension in 1 ml of PBS and centrifuged at 9.2×g for 5 min; the pellets were subsequently disaggregated and fixed with 4% paraformaldehyde in PBS via overnight incubation at 4 °C. Subsequently, the samples were washed as before and disaggregated in 50 µl of PBS, and 1 µl of FITC-conjugated rat anti-mouse IgA monoclonal antibody (BD Pharmingen, BD Biosciences) was added. The samples were incubated for 20 min at room temperature and washed with PBS. The samples were resuspended in 400 µl of PBS, and 5 µL of propidium iodide (PI) (BD Pharmingen, BD Biosciences) were added before analysis by flow cytometry. Sample analysis was conducted with a BD FACSAria Fusion Flow Cytometer (BD 400, BD Biosciences) by using BD FACSDiva v.8.0.2 acquisition and analysis software (BD Biosciences, Microsoft Windows 10). In total, 10,000 events were acquired from the gate, as shown in the forward scatter (FSC)/side scatter (SSC) dot plot. The percentage (%) of IgA–microbiota complexes was determined based on events with double-positive staining (IgA–FITC+/PI+). Double-negative (PI−/IgA−) or single-positive (PI−/IgA+ and PI+/IgA−) events were excluded from analysis.

### Relative mRNA expression of mucins and IL-18

The relative expression of *MUC2, MUC5AC* and *interleukin-18* (*IL-18*) mRNA was evaluated by RT–qPCR, and mRNA was extracted from whole mucosa samples with TRIzol (TRI Reagent RT, Molecular Research Center) according to the manufacturer’s instructions.

Complementary DNA (cDNA) synthesis was performed using a cDNA Synthesis Kit (Bio-Rad Laboratories) according to the manufacturer’s instructions. qPCR was performed in a thermal cycler (Mastercycler gradient, Eppendorf, Brinkmann Instruments) under the following conditions: priming for 5 min at 25 °C, reverse transcription for 20 min at 46 °C, and reverse transcription inactivation for 1 min at 95 °C. The obtained cDNA was stored at −20 °C for further analysis of mucin and cytokine gene expression.

The primer sequences for *MUC2, MUC5AC, IL-18* and glyceraldehyde 3-phosphate dehydrogenase (*GAPDH*), as a control gene, were designed using genomic information available from the National Center for Biotechnology Information (NCBI) with the Primer Express program v.3.0.1 (Table 1).

**Table 1:** Primer sequences for the RT-qPCR assay

Gene	Forward (5´→3´)	Temperature (°C)	Reverse (5´→3´)	Temperature (°C)
*GAPDH*	GAT GCC CCC ATG TTT GTG AT	55.8	GGT CAT GAG CCC TTC CAC AAT	57.0
*MUC2*	GCT GTG TGC CCT TGG CTA AG	59.0	ATT GAC AGG TGT GGC CAA TCA	57.1
*MUC5AC*	GTG ATG CAC CCA TGA TCT ATT TTG	54.4	CTG CCA CCA GCC CAT TG	57.0
*IL-18*	AAA GAA AGC CGC CTC AAA CC	57.4	TTC CAG GTC TCC ATT TTC TCC	54.7

For PCR, SsoAdvanced Universal SYBR Green Supermix (Bio-Rad Laboratories) was used according to the manufacturer’s instructions. The Applied Biosystem Step One Real-Time PCR System (Step One system, Life Technologies) was used with the following conditions: 1 cycle of 95 °C for 10 min, 40 cycles of 95 °C for 15 s and 60 °C for 23 s and 1 cycle of 95 °C for 15 s, 60 °C for 1 min and 95 °C for 15 s. The results are expressed as the 2^ΔΔ CT^ of each sample [16].

### Statistical analysis

For statistical analysis, data from ten samples per group (histological and flow cytometry) or from six samples per group (RT–qPCR) were included. The results that fit the normality test were analyzed with the parametric Student’s *t*-test; otherwise, for data that did not fulfill the normality test, the nonparametric Mann‒Whitney *U* or Wilcoxon test were applied. The data were analyzed using GraphPad Prism statistical software v.8.0.1 (GraphPad Software). The results analyzed by Student’s *t*-test were expressed as the mean ± s.e.m.; the data analyzed with Mann‒Whitney *U* or Wilcoxon tests were expressed as the median (midline), 1^st^ quartile (Q1) (bottom line), and 3^rd^ quartile (Q3) (top line); the minimum and maximum values; and the *z*-score. A *P* value ≤ 0.05 was considered to indicate statistical significance.

## RESULTS

### Stress increased the number of PAS-stained goblet cells only in the distal small intestine

**Figure 1:**
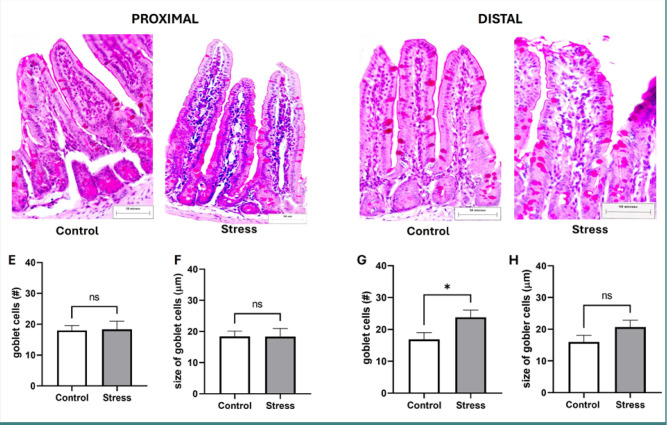
A–D, Histological analysis of goblet cells positive by PAS staining in the proximal and distal regions of the small intestine from control and stressed mice (×40 magnification). Representative images of goblet cell distribution in villi and crypts in the proximal intestinal regions of control (A) and stressed (B) mice and in the distal intestinal region of control (C) and stressed (D) mice. E–H, The number of goblet cells or villi stained with PAS (a marker of neutral mucins) in the proximal (E) and distal regions (G), and the size of the goblet cells (µm) in the proximal (F) and distal regions (H). The data represent the mean ± s.e.m. from two independent assays; *P < 0.05; ns, not significantly different (Student’s t-test).

Histological evaluation via PAS staining was performed to detect neutral mucins. Figure 1 shows histological images of the proximal intestinal region (Figure 1A,B) and distal intestinal region (Figure 1C,D) of the small intestine from control and stressed mice, as well as the number (Figure 1E,G) and size (Figure 1F,H)of goblet cells in both intestinal segments. The histological data (mean ± s.e.m.) indicated that, compared to those in the control group, the number and size of the goblet cells in the stressed mice were not significantly different in the proximal region (Figure 1E,F); however, in the distal region, stress led to a significant increase in the number of goblet cells (control 16.89 ± 2.09 vs. stress 23.80 ± 2.27, *P* = 0.0403; Figure 1G) without altering their size (Figure 1H).

### Stress increased the number of AB-stained goblet cells only in the distal small intestine

**Figure 2:**
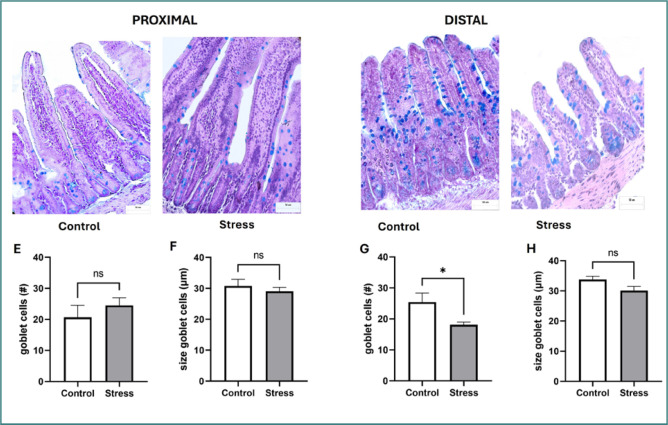
A–D, Histological analysis of goblet cells positive for AB staining in the proximal and distal regions of the small intestine from control and stressed mice (×40 magnification). Representative images of goblet cell distributions in villi and crypts in the proximal intestinal regions of control (A) and stressed (B) mice and in the distal intestinal region of control (C) and stressed (D) mice. E–H, The number of goblet cells or villi positive for AB (used as a marker of acid mucins) in the proximal (E) and distal regions (G), and the size of goblet cells (µm) in the proximal (F) and distal regions (H). The data represent the mean ± s.e.m. from two independent assays; *P < 0.05; ns, no significant difference (Student’s t-test).

Histological evaluation via AB staining was performed to detect acid mucins. Figure 2 shows histological images of the proximal intestinal region (Figure 2A,B) and the distal intestinal region (Figure 2C,D) of the small intestines of control and stressed mice. Histological data (mean ± s.e.m.) indicated that, with regard to the control group, the number and size of the goblet cells in the stressed mice did not significantly differ in the proximal region (Figure 2E,F); nevertheless, in the distal region, the number of goblet cells significantly decreased (control 25.40 ± 2.93 cells vs. stress 18.11 ± 0.87 cells, *P* = 0.0366; Figure 2G), and no changes in the size of the cells were observed (Figure 2H).

### Stress increased *MUC5AC* mRNA expression only in the proximal small intestine

**Figure 3:**
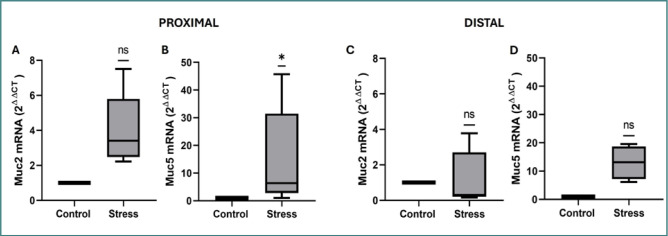
A–D, Relative *Muc2* mRNA expression in the proximal (A) and distal (C) regions and *Muc5ac* in the proximal (B) and distal (D) regions of the small intestine from control and stressed mice. The data in box and whisker formats show the median, 1^st^ quartile (Q1) (box bottom line), 3^rd^ quartile (Q3) (box top line), and minimum and maximum values (whiskers). *P < 0.05; ns, no significant difference (Wilcoxon’s test).

Analysis of mucins in the proximal region (Figure 3) demonstrated that *MUC2* mRNA expression was not significantly modified in the stressed group compared with the control group (Figure 3A), but *MUC5AC* mRNA expression was significantly increased (*z*-score = −2.36, *P* = 0.0156; Figure 3B). In the distal region, the expression of *MUC2* and *MUC5AC* mRNAs was unaffected in the stressed mice compared with the control group (Figure 3C,D). The quantitative results of all the groups (*z*-score, *P* value, median, maximum and minimum values) are shown in Table 2.

**Table 2:** Relative mRNA expression of mucins and IL-18 in the proximal and distal intestinal segments

Proximal intestinal segment				
	Median (range)		*z*-score	*P* value
	Control	Stress		
*MUC2*	1 (1–1)	3.41 (2.22–7.51)	−2.026	0.0625
*MUC5AC*	1 (1–1)	6.36 (1.03–45.74)	−2.360	0.0156
*IL-18*	1 (1–1)	1.79 (1.05–3.07)	−2.360	0.0115
**Distal intestinal segment**				
	**Median (range)**		**z-score**	***P* value**
	**Control**	**Stress**		
*MUC2*	1 (1–1)	0.30 (0.16–3.78)	−0.169	0.9375
*MUC5AC*	1 (1–1)	13.0 (6.15–19.57)	−2.022	0.0625
*IL-18*	1 (1–1)	1.15 (0.61–3.05)	−1.014	0.2136

### Stress increased *IL-18* mRNA expression only in the proximal small intestine

**Figure 4:**
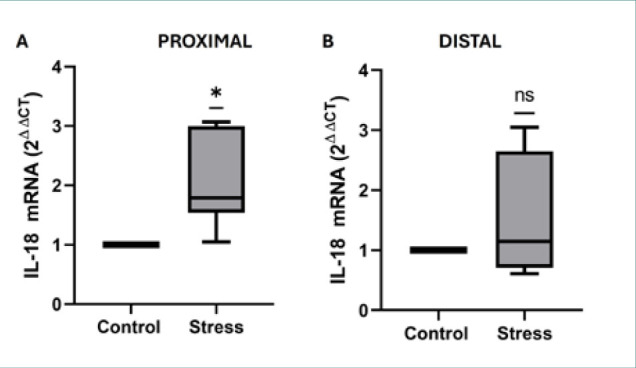
A,B, Relative mRNA expression of proinflammatory IL-18 in the proximal (A) and distal (B) regions. The data in box and whisker formats show the median, 1^st^ quartile (Q1) (box bottom line), 3^rd^ quartile (Q3) (box top line), and minimum and maximum values (whiskers). *P < 0.05; ns, no significant difference (Wilcoxon’s test).

Analysis of *IL-18* mRNA expression (Figure 4) showed that, compared with those in the control group, the IL-18 mRNA levels in the stressed mice were greater in the proximal region (*z*-score = −2.36, *P* = 0.0115; Figure 4A), but an apparent increase was observed in the distal region (Figure 4B). The quantitative results of all the groups (*z*-score, *P* value, median, maximum and minimum values) are shown in Table 2.

### Stress decreased the percentage of IgA–microbiota complexes only in the proximal small intestine

**Figure 5:**
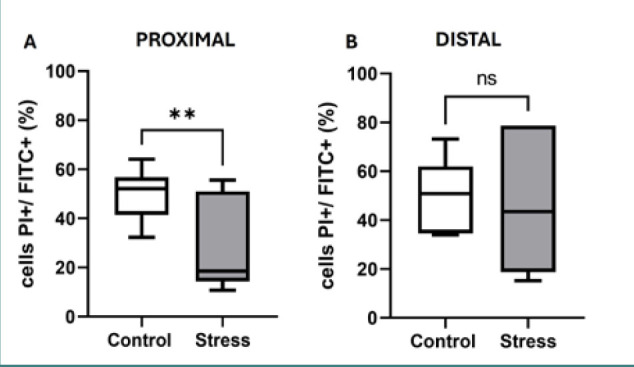
A,B, Percentage (%) of IgA complexes in the proximal (A) and distal (B) regions. The data in box and whisker formats show the median, 1^st^ quartile (Q1) (box bottom line), 3^rd^ quartile (Q3) (box top line), and minimum and maximum values (whiskers). *P < 0.05; ns, no significant difference (Mann-Whitney U-test).

To evaluate the effect of stress on the formation of IgA–microbiota complexes, flow cytometry analysis was performed (Figure 5). The results showed a lower percentage of IgA–microbiota complexes in the proximal region in stressed mice compared to the control group (*z*-score = 2.61, *P* = 0.0065) (Figure 5A). Nonetheless, in stressed mice, no significant differences in the percentage of complexes were found with respect to the control group in the distal region (Figure 5B).

## DISCUSSION

According to these findings, in this study, we revealed the differential effects of stress on biomarkers in the proximal versus distal small intestine. These findings may reflect the regionalized distribution of neurotransmitters and stress hormones derived from neuronal and non-neuronal cells that drive regulatory pathways [4,9,10,17]. Data from the distal region of the small intestine showed that chronic stress increased the number of goblet cells that were positively stained with PAS to detect neutral mucins; these findings may reflect an early transitory increase in the goblet cell count. This assumption is based on kinetic assays in which stress reduced the number of the PAS+ goblet cells; however, mucin levels increased after 3 days post stress but decreased thereafter 5 days post stress, as documented in the colons of rats that underwent chronic restraint stress [18].

Chronic stress induced a decrease in the number of goblet cells stained with AB in the distal segment; this stain was used to visualize acidic mucins. Stress leads to a decrease in the number of AB+ goblet cells in the ileum of mice under restraint stress [12]. The presumed mechanism through which stress reduces the number of goblet cells involves both stress-induced muscarinic signaling pathway activation and the release of mediators from stress-induced mast cell degranulation [18]. Some muscarinic receptors provide signals that inhibit goblet cell turnover by blocking the differentiation and proliferation of pluripotential Lgr5+ stem cells located at the crypt base [19,20]. Interestingly, the impact of stress on reducing goblet cell numbers involves suppression of the Notch pathway; the latter determines the differentiation of components of the epithelial monolayer from stem cells [21].

The present study revealed that stress induced an apparent increase in *MUC2* mRNA in the proximal small intestine without affecting *MUC2* mRNA in the distal region. As previously documented in mice, chronic restraint stress induces an apparent increase in *MUC2* mRNA levels in the duodenum or a decrease in the ileum [12]. *MUC2* is expressed throughout the entire length of the intestinal tract and is critical to intestinal homeostasis [4]. The divergent effects of stress on goblet cells and *MUC2* expression in the proximal and distal regions of the small intestine may result from muscarinic receptors that exhibit regionalized expression in the intestinal tract and provide signals that differentially modulate goblet cell turnover [19,20].

Although the effect of stress on upregulated *MUC2* RNA expression in the proximal segment was apparent in the present study, increased *MUC2* mRNA expression may reflect a protective response against the proinflammatory environment induced by stress; this idea is based on stress-induced increases in the *IL-18* mRNA level only in the proximal region. Previous studies involving mice subjected to long-term isolation indicated that stress induced a decrease in *MUC2* mRNA along with an increase in *IL-18* mRNA in the rectum; furthermore, stress upregulated *MUC2* mRNA expression, although it also increased *IL-18* mRNA levels in the colon [22]. Thus, the interplay between *MUC2* mRNA and *IL-18* mRNA in the proximal small intestine needs to be addressed in future assays.

*MUC5AC* is prominently expressed in the gastric mucosa, where it plays a protective role [17]. In experimental models of acute stress, *MUC5AC* mRNA expression was decreased in the gastric mucosa [23], but acute or chronic stress did not affect *MUC5AC* mRNA in any region of the small intestine [12,24]. In the present study, chronic stress upregulated *MUC5AC* mRNA expression only in the proximal segment. Increased *MUC5AC* expression in the intestine is regarded as a mechanism of protection under conditions of intestinal inflammation [25]. In this work, the parallel increase in *IL-18* and *MUC5AC* mRNA levels only in the proximal small intestine may suggest an underlying inflammatory response induced by stress. *MUC5AC* mRNA expression can be upregulated in some diseases in which stress is a contributing factor to inflammatory pathologies such as Crohn’s disease [26], which affects the proximal small intestine, although most commonly the ileum and colon [27].

Considering their pivotal role in intestinal homeostasis [5–7], the analysis of IgA–microbiota complexes revealed that their abundance was reduced in the proximal small intestine or unaffected in the distal region following stress. The findings on IgA–microbiota generation may reflect the changes in the pattern of MUC2 glycosylation that drive the flattening and loss of adhesiveness for bacteria induced by stress [28,29]. Moreover, stress favors the direct contact of luminal bacteria with the epithelial surface, thus eliciting an inflammatory response, as has been documented in an experimental model of chronic stress in rats [30]. The proximal region of the small intestine contains a lower microbiota density [7]; therefore, in this region, IgA–microbiota complexes have a critical role in counteracting the inflammatory response induced by the direct interaction of bacteria with the epithelial cell monolayer [5]. According to the findings of this study, a decrease in IgA–microbiota complexes along with an increase in *MUC5AC* and *IL-18* mRNA expression suggests that stress promotes a proinflammatory environment in the proximal segment of the small intestine; the underlying mechanism may entail contact between the microbiota and the epithelial surface, which drives inflammatory pathways via innate Toll-like receptor signals. In this study, sampling of whole mucosal scrapings from proximal and distal regions to evaluate the outcome of stress on IgA–microbiota complexes associated with the epithelium was not assessed. This aspect may be addressed in a future contribution owing the divergent effect of stress on the microbiota located at the mucosal epithelium versus the luminal-associated microbiota [31].

## Conclusion

The effect of chronic stress on goblet cell cellularity occurred only in the distal segment and did not affect the proximal region; moreover, the impact of stress on upregulating both *MUC5AC* and *IL-18* mRNA expression or reducing the number of IgA–microbiota complexes suggested a prominent proinflammatory outcome in the proximal segment of the small intestine. These findings may provide an experimental reference for human diseases that can affect the proximal small intestine, such as Crohn’s disease, in which stress contributes to the progression of intestinal inflammation or relapse. Finally, the data provide insights into pharmacologic interventions to control the proinflammatory effect of stress on intestinal barrier dysfunction in the proximal and distal small intestine segments.

## Data Availability

Further data are available from the corresponding author upon reasonable request.

## Autorship

J.K.G.-G., R.O.-P., and M.G.-V. contributed to the acquisition and analysis of the data. F.G.-M. and M.E.D.-S. contributed to drafting the work and revised it critically for important intellectual content. J.K.G.-G., F.G.-M., M.G.-V., and M.E.D.-S. made substantial contributions to the conception or design of the work, interpretation of the data for the work, drafting of the work, and critical revision of the manuscript for important intellectual content. All authors have read and approved the final version of the manuscript.

## References

[1] Bischoff SC, Barbara G, Buurman W, Ockhuizen T, Schulzke JD, Serino M (2014). Intestinal permeability–a new target for disease prevention and therapy. BMC Gastroenterol.

[2] Cornick S, Tawiah A, Chadee K (2015). Roles and regulation of the mucus barrier in the gut. Tissue Barriers.

[3] Bowcutt R, Forman R, Glymenaki M, Carding SR, Else KJ, Cruickshank SM (2014). Heterogeneity across the murine small and large intestine. World J Gastroenterol.

[4] Pelaseyed T, Bergström JH, Gustafsson JK, Ermund A, Birchenough GMH, Schütte A (2014). The mucus and mucins of the goblet cells and enterocytes provide the first defense line of the gastrointestinal tract and interact with the immune system. Immunol Rev.

[5] DuPont HL, Jiang Z-D, Alexander AS, DuPont AW, Brown EL (2023). Intestinal IgA-Coated Bacteria in Healthy-and Altered-Microbiomes (Dysbiosis) and Predictive Value in Successful Fecal Microbiota Transplantation. Microorganisms.

[6] Hoces D, Arnoldini M, Diard M, Loverdo C, Slack E (2020). Growing, evolving and sticking in a flowing environment: understanding IgA interactions with bacteria in the gut. Immunology.

[7] Mowat AM, Agace WW (2014). Regional specialization within the intestinal immune system. Nat Rev Immunol.

[8] Liu Y, Yuan X, Li L, Lin L, Zuo X, Cong Y, Li Y (2020). Increased Ileal Immunoglobulin A Production and Immunoglobulin A-Coated Bacteria in Diarrhea-Predominant Irritable Bowel Syndrome. Clin Transl Gastroenterol.

[9] Sharkey KA, Mawe GM (2002). Neuroimmune and epithelial interactions in intestinal inflammation. Curr Opin Pharmacol.

[10] He J, Guo H, Zheng W, Yao W (2018). Effects of Stress on the Mucus-microbial Interactions in the Gut. Curr Protein Pept Sci.

[11] Lin R, Wang Z, Cao J, Gao T, Dong Y, Chen Y (2020). Role of melatonin in intestinal mucosal injury induced by restraint stress in mice. Pharm Biol.

[12] Habiyambere B, Onyango E (2016). Chronic Stress Modulates the Mucin Components of the Intestinal Barrier and the Intestinal Morphology. Br J Med Med Res.

[13] Zhang Y, Wu S, Liu Y, Ma J, Li W, Xu X (2021). Acute Cold Water-Immersion Restraint Stress Induces Intestinal Injury and Reduces the Diversity of Gut Microbiota in Mice. Front Cell Infect Microbiol.

[14] Machorro-Rojas N, Sainz-Espuñes T, Godínez-Victoria M, Castañeda-Sánchez JI, Campos-Rodríguez R, Pacheco-Yepez J (2019). Impact of chronic immobilization stress on parameters of colonic homeostasis in BALB/c mice. Mol Med Rep.

[15] van der Waaij LA, Mesander G, Limburg PC, van der Waaij D (1994). Direct flow cytometry of anaerobic bacteria in human feces. Cytometry.

[16] Livak KJ, Schmittgen TD (2001). Analysis of relative gene expression data using real-time quantitative PCR and the 2-ΔΔCT method. Methods.

[17] Larsson JMH, Thomsson KA, Rodríguez-Piñeiro AM, Karlsson H, Hansson GC (2013). Studies of mucus in mouse stomach, small intestine, and colon III Gastrointestinal Muc5ac and Muc2 mucin O-glycan patterns reveal a regiospecific distribution. Am J Physiol Gastrointest Liver Physiol.

[18] Pfeiffer CJ, Qiu B, Lam SK (2001). Reduction of colonic mucus by repeated short-term stress enhances experimental colitis in rats. J Physiol Paris.

[19] Greig CJ, Armenia SJ, Cowles RA (2020). The M1 muscarinic acetylcholine receptor in the crypt stem cell compartment mediates intestinal mucosal growth. Exp Biol Med.

[20] Uwada J, Nakazawa H, Muramatsu I, Masuoka T, Yazawa T (2023). Role of Muscarinic Acetylcholine Receptors in Intestinal Epithelial Homeostasis: Insights for the Treatment of Inflammatory Bowel Disease. Int J Mol Sci.

[21] Shigeshiro M, Tanabe S, Suzuki T (2012). Repeated exposure to water immersion stress reduces the Muc2 gene level in the rat colon via two distinct mechanisms. Brain Behav Immun.

[22] Nishida K, Kamizato M, Kawai T, Masuda K, Takeo K, Teshima-Kondo S (2009). Interleukin–18 is a crucial determinant of vulnerability of the mouse rectum to psychosocial stress. FASEB J.

[23] Choo D, Khwaja K, Nori K, Rewinski M, Zeff R, Perdrizet G (2002). In vivo characterization of the molecular-genetic changes in gastric mucosa during the development of acute gastritis and stress ulceration. J Trauma.

[24] Zhang Y, Duan C, Wu S, Ma J, Liu Y, Li W (2022). Knockout of IL-6 mitigates cold water-immersion restraint stress-induced intestinal epithelial injury and apoptosis. Front Immunol.

[25] Olli KE, Rapp C, Connell LO, Collins CB, McNamee EN, Jensen O (2020). Muc5ac expression protects the colonic barrier in experimental colitis. Inflamm Bowel Dis.

[26] Ge L, Liu S, Li S, Yang J, Hu G, Xu C (2022). Psychological stress in inflammatory bowel disease: Psychoneuroimmunological insights into bidirectional gut–brain communications. Front Immunol.

[27] Buisine MP, Desreumaux P, Leteurtre E, Copin MC, Colombel JF, Porchet N (2001). Mucin gene expression in intestinal epithelial cells in Crohn’s disease. Gut.

[28] Da Silva S, Robbe-Masselot C, Ait-Belgnaoui A, Mancuso A, Mercade-Loubière M, Salvador-Cartier C (2014). Stress disrupts intestinal mucus barrier in rats via mucin O-glycosylation shift: Prevention by a probiotic treatment. Am J Physiol Gastrointest Liver Physiol.

[29] Da Silva S, Robbe-Masselot C, Raymond A, Mercade-Loubière M, Salvador-Cartier C, Ringot B, Léonard R, Fourquaux I, Ait-Belgnaoui A, Loubière P (2015). Spatial localization and binding of the probiotic Lactobacillus farciminis to the rat intestinal mucosa: Influence of chronic stress. PLoS One.

[30] Söderholm JD, Perdue MH (2001). Stress and the gastrointestinal tract II. Stress and intestinal barrier function. Am J Physiol Gastrointest Liver Physiol.

[31] Galley JD, Yu Z, Kumar P, Dowd SE, Lyte M, Bailey MT (2014). The structures of the colonic mucosa-associated and luminal microbial communities are distinct and differentially affected by a prolonged murine stressor. Gut Microbes.

